# Minimal residual disease in diffuse large B‐cell lymphoma after first‐line therapy: Defining the continuum from metabolic remission to molecular clearance

**DOI:** 10.1111/bjh.70619

**Published:** 2026-06-14

**Authors:** Santino Caserta, Enrica Antonia Martino, Ernesto Vigna, Antonella Bruzzese, Mamdouh Skafi, Nicola Amodio, Eugenio Lucia, Virginia Olivito, Caterina Labanca, Francesco Mendicino, Maria Eugenia Alvaro, Fortunato Morabito, Massimo Gentile

**Affiliations:** ^1^ Hematology Unit, Department of Onco‐Hematology AO of Cosenza Cosenza Italy; ^2^ Emergency and Internal Medicine Department Saint Joseph Hospital East Jerusalem Palestine; ^3^ Department of Experimental and Clinical Medicine University of Catanzaro Catanzaro Italy; ^4^ AIL Sezione di Cosenza Cosenza Italy; ^5^ Department of Pharmacy, Health and Nutritional Science University of Calabria Rende Italy

**Keywords:** circulating tumour DNA, clonal persistence, diffuse large B‐cell lymphoma, FDG‐PET, minimal residual disease, relapse, response assessment

## Abstract

Response assessment in diffuse large B‐cell lymphoma (DLBCL) has traditionally relied on anatomical and metabolic imaging, with fluorodeoxyglucose positron emission tomography (FDG‐PET)–defined complete metabolic remission representing the principal end‐point of first‐line therapy. However, growing evidence indicates that metabolic control does not necessarily correspond to clonal eradication. Circulating tumour deoxyribonucleic acid (ctDNA)–based minimal residual disease (MRD) assessment offers a complementary perspective by detecting systemic clonal persistence rather than localized tumour burden. In this review, we synthesize biological, methodological and clinical evidence showing that PET and MRD capture distinct and only partially overlapping dimensions of treatment response. We discuss biological implications of PET‐negative/MRD‐positive status, which may represent occult residual disease driven by subthreshold metabolic activity, diffuse low‐volume dissemination or microenvironment‐mediated resistance. MRD may therefore refine prognostic stratification beyond PET, particularly among patients achieving complete metabolic responses. Nevertheless, technical variability, limited standardization and insufficient prospective validation currently limit its use as a routine therapeutic decision trigger. Emerging frameworks integrating PET and MRD, especially within adaptive clinical trials, conceptualize response as a dynamic biological process rather than a static classification. Overall, metabolic remission likely reflects control of dominant tumour compartments, whereas MRD detection identifies persisting therapy‐resistant clones. Integrating both dimensions may improve interpretation and guide therapeutic strategies.

## INTRODUCTION

Response assessment in diffuse large B‐cell lymphoma (DLBCL) has traditionally relied on radiological and metabolic criteria, with fluorodeoxyglucose positron emission tomography (FDG‐PET)–defined complete metabolic remission (CMR) representing the principal surrogate of therapeutic success in both clinical practice and clinical trials.^1^, ^2^ However, accumulating evidence indicates that radiological and metabolic end‐points do not fully capture the biological state of residual disease.^3^

Standard imaging–based response criteria are inherently limited in their ability to resolve low‐volume or biologically quiescent malignant clones.^4^ Complete metabolic response reflects suppression of glycolytic activity and reduction of measurable tumour mass, but does not necessarily imply eradication of the malignant clone.^5^

Imaging and molecular assays must be interpreted in the light of their diagnostic performance. In DLBCL, end‐of‐treatment (EOT) FDG‐PET/computed tomography (CT) has a high negative predictive value for relapse, whereas its positive predictive value is more variable due to inflammatory or treatment‐related uptake. In contrast, circulating tumour deoxyribonucleic acid (ctDNA)–based minimal residual disease (MRD) assays offer high analytical sensitivity for molecular persistence but do not always correlate with clinical relapse. These differences indicate that both modalities provide probabilistic, rather than definitive, evidence of disease eradication. Considering sensitivity, specificity and predictive values supports viewing PET and MRD as complementary tools assessing distinct biological dimensions of treatment response.

These observations motivate a conceptual shift in response assessment: MRD should be regarded not as a measure of residual tumour bulk, but as a marker of persistent malignant clonality. Several reviews have outlined the technical landscape and early clinical applications of MRD assays in DLBCL, particularly ctDNA‐based approaches and their prognostic significance. Our aim here is not to recapitulate these overviews, but to extend them by focusing specifically on the biological interpretation of PET/MRD discordance and on a temporally structured framework of clonal dynamics during front‐line therapy.^6^

Building on these prior reviews, we focus specifically on (i) the biological interpretation of PET/MRD discordance; (ii) a temporal framework for MRD kinetics during front‐line therapy^6^; and (iii) how these concepts may—and may not—inform clinical decision‐making.

Importantly, the objective of molecular monitoring in this context is not surveillance for overt clinical relapse, but the identification of early therapeutic failure. MRD detection is considered here as a tool to reveal insufficient clonal eradication at a subclinical stage, when conventional imaging suggests a complete response but residual malignant clones remain biologically active. This distinction is critical, as it reframes MRD not as a post‐remission monitoring strategy, but as a means to refine response assessment and risk stratification during initial therapy.^7^

## BIOLOGICAL BASIS OF MOLECULAR PERSISTENCE IN DLBCL

### Clonal heterogeneity and evolution under first‐line therapy

DLBCL exhibits marked intratumoural genetic heterogeneity, with coexisting subclones differing in genomic alterations, therapy sensitivity and metastatic potential.^8^ Multiregion sequencing demonstrates spatial heterogeneity, showing genetically distinct populations occupying different anatomical sites within the same tumour, which limits the representativeness of single‐site biopsies and contributes to discordant treatment responses (e.g. Jaccard similarity variance across biopsies).^9^

This diversity provides the substrate for Darwinian selection during first‐line immunochemotherapy. Treatment eliminates therapy‐sensitive clones, while resistant lineages—often harbouring alterations in tumour suppressors (e.g. tumor protein p53 [TP53], lysine methyltransferase 2D [KMT2D]) and survival pathways—persist and expand. Studies of relapsed/refractory DLBCL demonstrate that mutations affecting TP53 and histone modifiers can remain clonally persistent at relapse, implicating them in primary resistance.^10^, ^11^

The molecular heterogeneity of DLBCL should be considered when interpreting MRD dynamics. Cell‐of‐origin (COO) classification distinguishes germinal centre B‐cell–like (GCB) and activated B‐cell–like (ABC/non‐GCB) subtypes, while genomic frameworks further define subgroups such as myeloid differentiation primary response 88 (MYD88)/cluster of differentiation 79B (CD79B)‐mutated (MCD), B‐cell lymphoma 6 (BCL6) fusion/NOTCH2‐mutated (BN2), NOTCH1‐mutated (N1) and enhancer of zeste homolog 2 (EZH2)‐mutated/B‐cell lymphoma 2 (BCL2)‐translocated (EZB). These categories reflect distinct oncogenic programmes that may influence MRD kinetics, ctDNA shedding and concordance between molecular and metabolic responses. ABC‐type DLBCL, often characterized by MYD88 and CD79B co‐mutations, provides stable molecular targets for ctDNA‐based MRD monitoring. In contrast, subtype‐specific differences in proliferation, tumour microenvironment and extra‐nodal spread may affect ctDNA detectability, contributing to variability in PET/MRD concordance.^12^

ctDNA reflects this evolutionary by capturing shifts in clonal populations throughout therapy. Baseline and on‐treatment ctDNA genotyping identifies dominant immunoglobulin gene rearrangements and subclonal variants associated with progression‐free survival (PFS) and overall survival (OS). Persistent ctDNA after several treatment cycles or at the end of treatment identifies patients at high risk of early progression, even among those achieving PET‐defined responses, revealing a molecular reservoir of resistant disease undetected by imaging.^12^

Systematic analyses further show that ctDNA dynamics improve risk stratification beyond PET‐CT, with MRD positivity predicting inferior outcomes irrespective of metabolic remission status. These findings support MRD as an indicator of persistent clonality rather than simply tumour burden.^13^

These patterns arise from therapy‐induced clonal shifts and resistant molecular niches, where subclones within protective microenvironments evade immune and cytotoxic pressure. The emergence of such niches during immunochemotherapy underscores the need for integrated molecular monitoring to reveal subclinical persistence.^14^

### Why complete metabolic remission may not reflect clonal clearance

CMR on FDG‐PET is conventionally interpreted as tumour eradication. However, CMR primarily reflects the suppression of glycolytic activity in macroscopic lesions rather than true clonal extinction. FDG uptake depends on glucose transporter expression and hexokinase‐mediated trapping, processes linked to proliferative and anabolic states. Consequently, PET measures the metabolic phenotype of dominant tumour populations but does not directly assess clonal viability, explaining frequent discordance between metabolic remission and molecular persistence in DLBCL.^15^

One explanation involves anatomical sanctuary sites and the intrinsic sensitivity limits of FDG imaging. Residual lymphoma cells may persist in compartments such as bone marrow, spleen or selected extra‐nodal tissues, where physiological background uptake and partial‐volume effects reduce the detectability of low‐volume disease. Diffuse malignant cells may also generate signals below the PET spatial resolution, producing apparent metabolic remission despite biological persistence. In addition, metabolic reprogramming under therapy may shift lymphoma cells from aerobic glycolysis to oxidative phosphorylation or alternative substrates, reducing FDG avidity without eliminating clonogenic potential. This metabolic plasticity produces genetically malignant but metabolically silent residual disease, a limitation highlighted in imaging and lymphoma biology studies.^7^

A second mechanism is the survival of low‐proliferative residual clones. Immunochemotherapy preferentially eradicates rapidly cycling, glycolytic cells, while subclones with reduced proliferative drive or enhanced stress tolerance persist. These cells often exhibit attenuation of MYC‐driven programmes and activation of pro‐survival pathways, including BCL2‐ and nuclear factor kappa‐light‐chain‐enhancer of activated B cells (NF‐κB)‐dependent signalling. Functionally, they resemble dormant reservoirs—genetically malignant but metabolically quiescent and poorly glycolytic—generating minimal PET signal. Genomic analyses demonstrate that relapse frequently originates from ancestral clones already present at diagnosis, rather than new lineages, indicating that resistant subclones persist silently before re‐expansion.^16^ In this framework, CMR reflects elimination of dominant metabolic phenotypes, whereas clonal persistence represents the survival of less proliferative therapy‐resistant lineages.

Additional discordance derives from the biology of ctDNA shedding. ctDNA does not directly measure tumour mass but reflects the balance between tumour cell survival and death, influenced by vascular access and microenvironmental context. Actively proliferating or apoptotic cells contribute disproportionately to ctDNA levels, whereas quiescent or stromally protected clones may shed DNA intermittently.^17^ Thus, ctDNA positivity in PET‐negative patients indicates ongoing malignant cell turnover, despite insufficient metabolic activity to generate detectable FDG signals. Conversely, clones embedded in supportive niches may persist with minimal ctDNA release, underscoring that PET and ctDNA probe different biological dimensions of disease burden. The capacity of ctDNA to capture clonal persistence, invisible to imaging, has been demonstrated in aggressive lymphomas.^18^

Overall, CMR signifies suppression of macroscopic FDG‐avid disease but does not guarantee extinction of all malignant clones, whereas ctDNA captures systemic clonal persistence through the dynamics of cell survival and death.

## METHODOLOGICAL APPROACHES TO MRD ASSESSMENT IN DLBCL

### Circulating tumour DNA quantification

The clinical translation of ctDNA‐based MRD assessment in DLBCL depends on how ctDNA is quantified and interpreted. Methodological choices influence analytical sensitivity and the biological meaning of a positive or negative result. Conceptually, ctDNA assays can be defined along two axes: tumour‐informed versus tumour‐agnostic design; and quantitative burden estimation versus qualitative detection of clonal persistence.

Tumour‐informed assays track patient‐specific molecular markers identified from diagnostic tumour tissue. By anchoring detection to known tumour‐derived sequences, these approaches achieve high analytical sensitivity and specificity, often reaching detection limits below 10^−5^, and are resistant to confounding by clonal haematopoiesis or background noise.^16^, ^18^ They are particularly suited for longitudinal monitoring to determine whether the original malignant clone persists after therapy. However, tumour‐informed assays require diagnostic tissue and additional sequencing steps, which increase turnaround time and cost and may limit routine scalability.

Tumour‐agnostic assays instead analyse plasma for recurrently mutated genes or lymphoma‐associated genomic regions without prior tumour sequencing. These strategies are operationally simpler and can be applied immediately at baseline, making them attractive for risk stratification and early response assessment. Their sensitivity is intrinsically limited by the need to distinguish tumour‐derived variants from sequencing artefacts and clonal haematopoiesis.^19^ Consequently, tumour‐agnostic approaches often detect ctDNA primarily when tumour burden or turnover is sufficiently high.^7^, ^20^ In practice, they are well suited to capturing early molecular response (EMR) kinetics but are less reliable for defining true MRD negativity at the end of treatment.

Another methodological dimension concerns whether ctDNA is interpreted quantitatively or qualitatively. Quantitative approaches measure ctDNA as variant allele frequency, haploid genome equivalents per millilitre or log‐fold reduction from baseline. These metrics correlate with total tumour burden and treatment response, and allow modelling of molecular response kinetics. Early ctDNA log‐reduction during therapy predicts outcome independently of imaging and reflects chemosensitivity.^21^, ^22^ Quantitative metrics are therefore most informative during treatment, when ctDNA levels change rapidly with cytoreduction.

At the end of treatment, the biological meaning of ctDNA shifts from burden estimation to detection of clonal survival. In this setting, qualitative detection becomes more relevant than absolute quantity. Even minimal tumour‐specific ctDNA implies persistence of malignant cells and incomplete clonal clearance. This distinction parallels the difference between tumour debulking and eradication: quantitative decline reflects reduction of dominant disease, whereas qualitative persistence indicates survival of residual clones. Accordingly, EOT MRD is best interpreted as a binary biological state—clonal present versus absent—since even very low‐level positivity is associated with high relapse risk.^18^

A further challenge is balancing analytical sensitivity and biological interpretability. Increasing sequencing depth and error correction allows detection of extremely low allele frequencies, but greater sensitivity does not automatically translate into clinical relevance. Ultra‐deep sequencing may identify DNA realized from dying tumour cells, treatment‐related DNA fragments or transient subclones with limited persistence. Thus, a technically positive signal does not represent biologically meaningful MRD. Conversely, clinically relevant residual clones may evade detection if metabolically quiescent, spatially sequestered or protected within microenvironmental niches.

Recent hybrid‐capture–based technologies illustrate advances in sensitivity. Cancer personalized profiling by deep sequencing (CAPP‐Seq) targets recurrent single‐nucleotide variants and achieves detection limits around 10^−4^ (∼100 ppm), although relapse may remain undetected at extremely low tumour fractions. Phase‐corrected error‐corrected deep sequencing (PhasED‐Seq) exploits phased variants to enhance error suppression and has detected MRD below 1 part per 10^6^, identifying ctDNA in cases negative by conventional CAPP‐Seq and anticipating PET‐defined relapse by several months.^6^

These considerations emphasize that ctDNA should not be treated solely as an analytical variable but within a biological and temporal framework. During therapy, quantitative ctDNA decline informs chemosensitivity and bulk tumour clearance. After therapy, qualitative persistence captures clonal survival. Across both phases, sampling timing and dynamic changes are as important as absolute ctDNA values. Methodological rigour therefore requires selecting assays according to the clinical question—estimating tumour burden, defining molecular response depth or establishing clonal eradication.

Different analytical platforms offer complementary trade‐offs between sensitivity, breadth and cost. Droplet digital polymerase chain reaction (ddPCR) provides inexpensive and rapid detection of predefined variants with sensitivity around 0.1%, but it is limited to known mutations and may be affected by leucocyte DNA contamination. Amplicon‐based next‐generation sequencing (NGS) expands coverage to multiple hotspot regions with sensitivities near 0.01% variant allele frequency, although it remains constrained to selected targets and requires robust error correction. Hybrid‐capture–based NGS further broadens genomic coverage and can reach sensitivity of 10^−4^–10^−5^, enabling simultaneous genotyping and MRD monitoring across large gene panels.^6^

**FIGURE 1:**
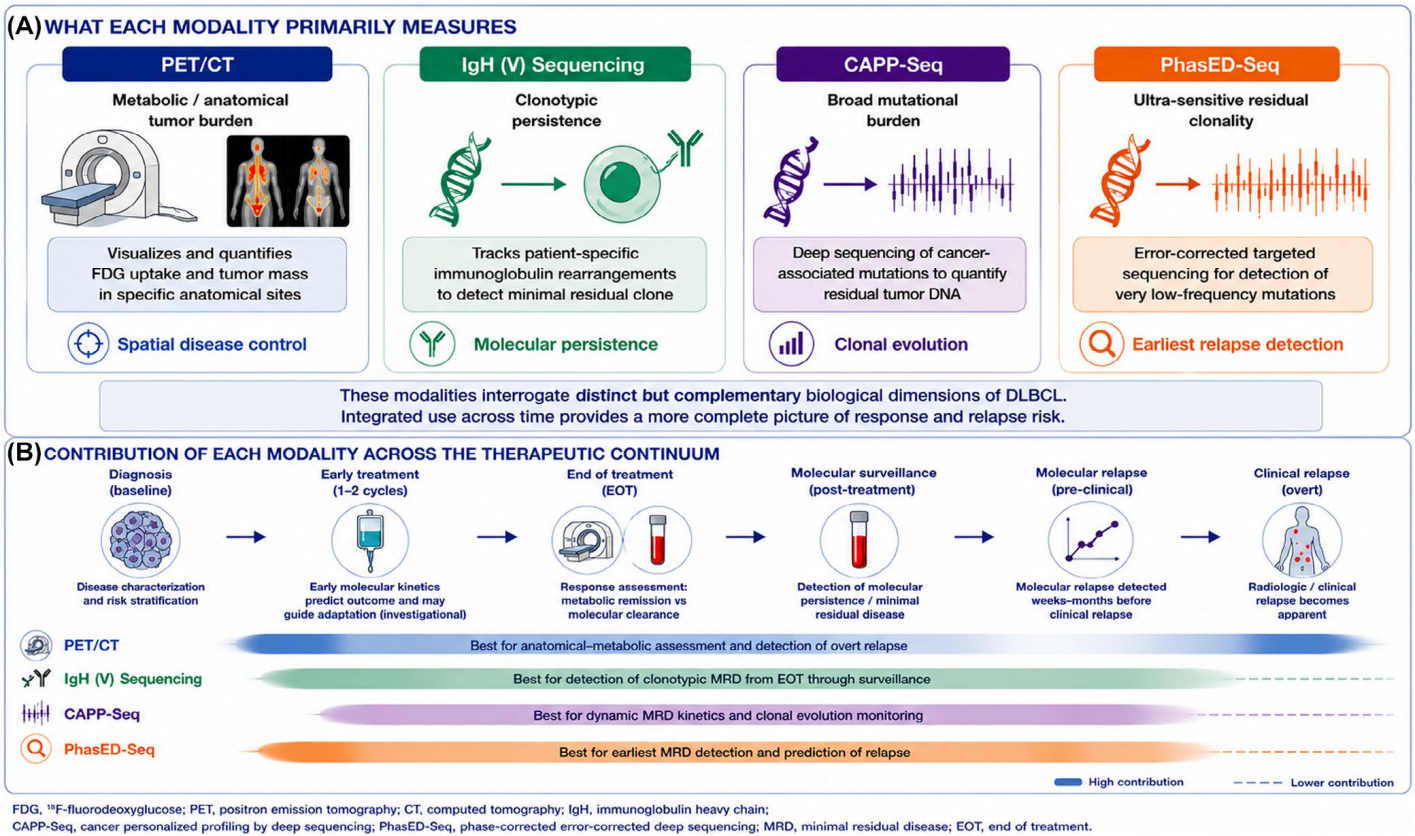
Complementary dimensions of metabolic and molecular response assessment in diffuse large B‐cell lymphoma (DLBCL). The figure illustrates the conceptual and temporal integration of major response assessment modalities in DLBCL. Panel A summarizes what each modality primarily measures: fluorodeoxyglucose positron emission tomography (FDG‐PET/CT) captures metabolic and anatomical tumour burden, while circulating tumour DNA (ctDNA)‐based approaches interrogate complementary molecular dimensions, including clonal persistence (IgH sequencing), broader mutational burden and clonal evolution (CAPP‐Seq) and ultra‐sensitive detection of residual disease and early relapse (PhasED‐Seq). Panel B depicts the contribution of each modality across the therapeutic continuum, from baseline disease characterization through early treatment dynamics, end‐of‐treatment assessment, molecular surveillance and preclinical relapse detection. Together, these modalities provide complementary insights into spatial disease control, clonal persistence, evolutionary dynamics and early relapse biology, supporting an integrated framework for response assessment in DLBCL.

To provide an integrated conceptual framework for interpretation, Figure 1 summarizes the complementary biological dimensions and temporal applications of the principal modalities used in DLBCL. Rather than representing competing technologies, these approaches interrogate distinct but interrelated aspects of disease biology, including anatomical tumour burden, clonal persistence, mutational complexity and early relapse dynamics across the therapeutic continuum. This integrated view highlights the value of combining imaging‐ and ctDNA‐based strategies to achieve a more comprehensive characterization of treatment response and residual disease.

### Different types of possible markers

The most specific markers for MRD tracking are tumour‐specific clonotypic sequences, particularly immunoglobulin gene rearrangements: immunoglobulin heavy‐chain (IGH) and immunoglobulin kappa light‐chain (IGK) rearrangements. These sequences uniquely identify the malignant B‐cell clone and are therefore ideal for tumour‐informed MRD monitoring. Once the clonotype is defined from diagnostic tissue, the same sequence can be searched in plasma with very high sensitivity.^23^

A second major class of markers comprises somatic mutations in the lymphoma genome. These include recurrent driver or trunk mutations such as those affecting *TP53, MYD88, CD79B, EZH2, CREB binding protein, KMT2D* and caspase recruitment domain family member 11.^24^ When identified at diagnosis, these mutations can be tracked longitudinally in plasma as surrogates of clonal persistence. Biologically, they may capture both dominant and subclonal populations, providing insight into clonal evolution under therapy. Practically, however, careful discrimination from mutations arising from clonal haematopoiesis, especially for genes like *TP53* or DNA methyltransferase 3 alpha, which may also be mutated in non‐malignant haematopoietic stem cells.^25^

An illustrative example is the EuroClonality / BIOMED‐2 – Next Generation / Nomenclature and Diagnostic Consortium (EuroClonality‐NDC) capture panel, which simultaneously interrogates immunoglobulin/T‐cell receptor gene rearrangements, lymphoma‐associated translocations (e.g. BCL2, BCL6, MYC) and recurrent single‐nucleotide variants. In a prospective R‐CHOP (rituximab, cyclophosphamide, doxorubicin, vincristine and prednisone) cohort, this assay identified at least one molecular marker in 90% of baseline ctDNA samples, supporting both genotyping and MRD monitoring.^15^

A third category includes lymphoma‐associated mutational panels used in tumour‐agnostic assays. These do not require prior tumour sequencing but instead interrogate plasma for a predefined set of genes frequently mutated in DLBCL. These markers are less specific for MRD at very low levels but are valuable for baseline risk stratification and for capturing EMR during therapy. Biologically, they reflect overall tumour‐derived DNA rather than the persistence of a defined clone, making them better suited for burden estimation than for definitive clonal clearance.

## TEMPORAL DYNAMICS OF MRD DURING FIRST‐LINE THERAPY

### Baseline ctDNA as a surrogate of tumour burden

At diagnosis, ctDNA levels correlate closely with total metabolic tumour volume and clinical risk indices, supporting its role as a quantitative surrogate of systemic tumour burden. Baseline ctDNA integrates disease across nodal and extra‐nodal compartments, overcoming sampling bias inherent to single‐site biopsy and the spatial resolution limits of imaging.

Prospective studies in newly diagnosed DLBCL have shown that higher pretreatment ctDNA concentrations are independently associated with inferior PFS and OS, even after adjustment for International Prognostic Index (IPI) and PET‐derived parameters. Importantly, ctDNA reflects not only tumour mass but also biological turnover; elevated baseline levels may capture proliferative and apoptotic dynamics not directly inferred from imaging alone.^26^

In a capture‐based NGS study using EuroClonality‐NDC, a pretreatment ctDNA threshold of 2.5 log hematopoietic gene expression (hGE)/mL stratified patients by established surrogate markers of tumour burden: higher baseline ctDNA was significantly associated with elevated lactate dehydrogenase, advanced Ann Arbor stage and high‐risk IPI. Although the sample size limited multivariable power, there was a clear trend towards inferior 2‐year PFS in the high‐ctDNA group.^15^

These observations are consistent with a broader meta‐analysis showing that high baseline ctDNA across lymphoma cohorts, predominantly DLBCL, is associated with roughly two‐ to threefold increased risks of progression and death.^6^

### Early molecular response (after 1–2 cycles)

EMR, typically assessed after two or three cycles of R‐CHOP, represents one of the most reproducible and clinically informative time points for ctDNA evaluation. In the EuroClonality‐NDC series, a ≥2.5‐log reduction in ctDNA after two cycles of R‐CHOP—defined as major molecular response (MMR)—stratified patients into markedly different outcomes: 2‐year PFS was 76% in those achieving MMR versus 0% in those who did not. In line with these data, other cohorts have shown that similar log‐fold reductions after 1–2 cycles identify patients with superior outcomes.^6^

Most MRD data derive from R‐CHOP–based therapy, but first‐line DLBCL treatment is evolving. Polatuzumab vedotin combined with rituximab, cyclophosphamide, doxorubicin and prednisone (pola‐R‐CHP) has emerged as an alternative front‐line regimen for previously untreated patients with an IPI score ≥2 who are eligible for anthracycline‐based chemoimmunotherapy. As MRD frameworks and ctDNA kinetic models were developed in R‐CHOP–treated cohorts, their applicability to pola‐R‐CHP remains uncertain. Whether antibody–drug conjugates alter ctDNA clearance, MRD thresholds or the frequency of PET/MRD discordance is unknown and warrants further investigation.

Biologically, early ctDNA kinetics reflect the chemosensitivity of the dominant clonal population. Whereas interim PET assesses reduction in glycolytic activity of macroscopic lesions, early ctDNA decline measures systemic clonal cytoreduction. These processes are related but not identical: molecular kinetics may precede PET response and, in some cases, refine risk stratification among patients with similar metabolic findings.

Recent analyses further suggest that early molecular non‐response may identify biologically aggressive subgroups characterized by persistent trunk mutations and limited clonal contraction under therapy. Using PhasED‐Seq, interim MRD positivity after three cycles of front‐line immunochemotherapy**—**despite imaging responses**—**was associated with hazard ratios ≥5 for PFS and OS compared with MRD‐negative patients. This underscores that failure to achieve early molecular clearance identifies a biologically aggressive subset even among clinically responding patients.^6^ From a practical perspective, early ctDNA dynamics currently serve primarily as a research tool to inform the development of response‐adapted strategies, rather than as a driver of routine clinical decisions. Their integration into everyday practice therefore remains limited and context‐dependent. In this regard, the National Comprehensive Cancer Network (NCCN) guidelines acknowledge a potential, but restricted, role for ctDNA assessment in selected scenarios—such as patients with PET‐positive findings at the end of treatment in whom biopsy confirmation is not feasible—where it may provide complementary molecular information to support clinical judgement^26^ (Table 1).

**TABLE 1:** Proposal of temporal dynamics of MRD during first‐line therapy.

Time point	Biological meaning of ctDNA/MRD	Relationship with PET	Clinical interpretation
Baseline (diagnosis)	Surrogate of systemic tumour burden; integrates nodal and extra‐nodal disease and reflects clonal heterogeneity	Correlates with metabolic tumour volume but captures disseminated disease beyond imaging resolution	High baseline ctDNA identifies patients with higher risk biology
After 1–2 cycles	Reflects chemosensitivity of dominant clones and early clonal contraction	Partially concordant with interim PET; may anticipate metabolic response or identify discordant biology	Rapid log‐fold ctDNA decline indicates favourable response; insufficient reduction identifies patients at risk of primary refractory disease
End of treatment	Qualitative indicator of clonal eradication versus persistence	Frequently discordant: PET‐negative/MRD‐positive status reflects occult residual disease	MRD positivity predicts relapse independently of PET and redefines ‘complete metabolic response’ as biologically incomplete
Post‐treatment surveillance	Detects molecular relapse before anatomical or metabolic recurrence; reflects re‐expansion of resistant clones	Precedes PET relapse by weeks to months (lead time)	Identifies preclinical failure of clonal control

Abbreviations: ctDNA, circulating tumour DNA; MRD, minimal residual disease; PET, positron emission tomography.

### End‐of‐treatment MRD

EOT MRD represents a biologically distinct phase compared with early ctDNA kinetics. At this stage, ctDNA detection shifts from quantitative assessment of tumour burden to qualitative evaluation of clonal persistence.

Multiple studies have demonstrated that ctDNA positivity at EOT strongly predicts subsequent relapse, including among patients achieving CMR by PET. Even very low‐level molecular positivity carries adverse prognostic significance, indicating that incomplete clonal eradication—rather than residual tumour bulk—is the primary determinant of relapse risk. Across prospective cohorts summarized in recent reviews, EOT ctDNA positivity is associated with hazard ratios of 5–10 for PFS and OS, even among patients in PET‐defined CMR, emphasizing that residual MRD reflects biological failure of eradication rather than trivial residual.^6^

This distinction underscores a critical conceptual point: PET and MRD interrogate different biological dimensions of response. PET assesses suppression of metabolically active tumour masses within anatomical compartments, whereas MRD evaluates systemic clonal persistence via tumour‐derived DNA fragments. Consequently, a PET‐negative/MRD‐positive status reflects a biological discordance**—**apparent local metabolic control with ongoing systemic clonal survival.

From a practical standpoint, EOT MRD positivity should be interpreted as evidence of residual malignant clonality rather than minimal tumour burden. However, the optimal clinical management of MRD‐positive patients at EOT remains undefined, and therapeutic intervention based solely on EOT MRD should currently be restricted to clinical trials.^26^

### Post‐treatment molecular surveillance

After completion of therapy, serial ctDNA monitoring enables detection of molecular relapse before clinical or radiographic progression. Several cohorts have demonstrated a lead time advantage of molecular detection ranging from weeks to several months compared with the identification of imaging‐based relapse. In longitudinal immunoglobulin high‐throughput sequencing and CAPP‐Seq studies, ctDNA detected relapse a median of approximately 3.5 months—and in some series >6 months—before radiographic progression, with positive predictive values near 90% and negative predictive values approaching 98%.^6^

However, no prospective trial has yet demonstrated that intervening at the time of molecular relapse improves survival compared with treatment at overt clinical progression. Consequently, expert reviews currently consider pre‐emptive MRD‐guided therapy to be investigational.^6^

Importantly, post‐treatment ctDNA surveillance does not simply replicate imaging at higher sensitivity. Rather, it detects re‐expansion of therapy‐resistant clones at a subclinical stage. Molecular recurrence therefore reflects biological reactivation of clonal proliferation before the development of metabolically overt lesions.

Nevertheless, not all molecular relapses translate immediately into symptomatic or radiographically detectable disease, and the clinical utility of pre‐emptive intervention remains uncertain. The balance between early detection and overtreatment represents a central unresolved issue in post‐remission monitoring strategies.^26^, ^27^

## PET‐NEGATIVE/MRD‐POSITIVE STATUS: THE BIOLOGY OF OCCULT RESIDUAL DISEASE

PET‐negative/MRD‐positive status defines a discordant response in which patients achieve CMR by FDG‐PET yet retain detectable tumour‐derived DNA in plasma. This can occur at two critical time points: (i) at the end of treatment, when PET indicates apparent eradication but ctDNA persists; and (ii) during surveillance, when molecular relapse precedes radiographic or clinical progression. Rather than reflecting minimal residual burden, this pattern indicates persistent malignant clonality below imaging resolution. The discordance highlights a mismatch between anatomical–metabolic control and systemic clonal clearance. In aggressive lymphomas, this subgroup shows relapse kinetics closer to refractory disease than to true complete responders^18^, ^20^

FDG‐PET detects areas of increased glycolytic activity, forming macroscopic lesions. Residual lymphoma cells, however, may survive therapy by reducing glycolysis, shifting towards oxidative phosphorylation or entering a low‐proliferative state. This metabolic reprogramming suppresses FDG avidity without abolishing clonogenic potential. Thus, PET negativity may reflect metabolic quiescence rather than cell death. Persistent ctDNA suggests ongoing turnover of residual cells releasing tumour DNA while remaining metabolically silent in tissue. This explains PET/MRD divergence after cytotoxic pressure, when selection favours clones with lower metabolic activity and greater stress tolerance.^14^

Spatial dilution of residual disease represents another mechanism. Therapy‐resistant clones may persist as dispersed populations in bone marrow, spleen or microfoci across organs rather than forming masses. Such a distribution can generate detectable ctDNA through cumulative turnover without focal FDG uptake above the physiological background. Clinically, both early MMR and ≥66% reduction in ΔSUVmax (maximum standardized uptake value) at interim PET independently predict improved PFS, but their combination provides the most precise risk stratification. Patients achieving both favourable MMR and ΔSUVmax reduction demonstrate 2‐year PFS rates around 80%–85%, whereas those lacking either parameter show PFS rates approaching 0%–20%.^15^

These findings support a systemic rather than compartmental disease state and align with observations that molecular relapse frequently precedes localized radiographic recurrence. Relapse therefore likely originates from a widely distributed reservoir of resistant cells rather than regrowth of a single dominant lesion, challenging the assumption that post‐remission disease remains anatomically localized.^28^

Residual clones may also persist within the tumour microenvironment niches. Stromal supports, hypoxia and immune evasion can maintain lymphoma cells in a drug‐tolerant state characterized by reduced proliferation and metabolism but preserved self‐renewal. PET‐negative/MRD‐positive disease therefore reflects both intrinsic resistance and persistence of permissive ecological niches. ctDNA detection indicates that these populations are not entirely dormant but continue limited turnover and remain capable of re‐expansion once therapeutic or immune pressure declines.^29^

Across multiple cohorts, ctDNA monitoring detects relapse earlier than imaging. PET‐negative/MRD‐positive patients have relapse rates similar to those with metabolic persistence, whereas PET‐negative/MRD‐negative patients typically experience durable remissions. Importantly, MRD positivity anticipates relapse temporally, supporting the interpretation that MRD reflects biological failure of eradication rather than simply radiographic disease volume.^18^, ^20^

The interval between molecular and radiographic relapse—often weeks to months—constitutes a biologically informative lead‐time. During this phase, resistant clones expand but have not yet formed a macroscopic tumour. MRD positivity therefore marks the transition from dormancy to clonal re‐expansion. PET cannot detect this stage because metabolic signals require a critical mass of proliferating cells. The lead‐time advantage of ctDNA thus reflects a genuine biological window in which disease persists as a systemic, low‐volume but clonally coherent population.

Consequently, PET‐negative/MRD‐positive status should be interpreted as the molecular equivalent of incomplete remission. It reveals failure of clonal extinction despite apparent metabolic control and marks a trajectory towards relapse rather than a static residual state. Relapse should therefore be viewed as a gradual biological process first detectable at the molecular level and only later becoming anatomically evident.^30^

## CLINICAL IMPLICATIONS OF MRD POSITIVITY AFTER FIRST‐LINE THERAPY

### Prognostic impact beyond PET

MRD positivity after first‐line immunochemotherapy is consistently associated with a markedly increased risk of relapse, including among patients achieving FDG‐PET CMR. This reflects a qualitative effect: molecular persistence identifies disease biologically primed for relapse. ctDNA captures not just residual bulk but survival of therapy‐resistant clonotypes that drive progression. Relapse in MRD‐positive/PET‐negative patients is often early and aggressive, suggesting failure of clonal extinction rather than delayed regrowth.^18^

Multivariable analyses incorporating PET response, IPI and baseline molecular features demonstrate that MRD retains prognostic significance. PET and MRD therefore capture non‐overlapping dimensions: metabolic lesion control versus systemic clonal survival. MRD is not a refinement of imaging but a distinct biomarker of response depth, reflecting whether malignant lineages are eradicated or suppressed. Mechanistically, molecular persistence represents an adaptive state selected under therapy rather than disease missed by imaging.^14^, ^27^

Among patients achieving CMR, MRD positivity identifies a subgroup with outcomes closer to partial responders than true complete responders, supporting biological risk reclassification. CMR is thus heterogeneous, comprising molecularly cleared and molecularly persistent states. Integrating analyses confirm ctDNA‐based MRD as one of the strongest prognostic factors in DLBCL, often outperforming IPI, cell‐of‐origin and interim PET in multivariable models.^15^

Clinically, post‐treatment MRD should be considered a powerful adverse prognostic marker that complements FDG‐PET. PET‐negative/MRD‐negative patients likely achieve true biological complete response, whereas MRD‐positive patients—regardless of PET status—harbour incompletely eradicated disease with high relapse risk.

### Clinical decision‐making challenges

The detection of MRD after first‐line therapy creates tension between biological insight and clinical utility. Although MRD positivity identifies patients at high relapse risk, it does not define a clear therapeutic threshold for intervention. Clinical actionability depends on whether early treatment improves outcomes versus therapy at overt relapse—an unresolved question. Thus, MRD positivity should be interpreted as evidence of incomplete eradication rather than as a trigger for therapy escalation. Acting prematurely risks conflating prognostic with predictive value. Currently, MRD is more robust for risk stratification than for guiding intervention.

A key challenge is distinguishing transient or technical signals from true biological persistence. Ultra‐sensitive assays may detect ctDNA from dying cells, treatment‐related shedding or minor subclones lacking long‐term potential. Conversely, relevant residual clones may shed DNA intermittently or at low levels, particularly within protective niches. MRD should therefore be viewed as a probabilistic, not binary, signal dependent on timing, dynamics and assay design. Persistent or rising ctDNA across serial samples is more indicative of true disease than a single low‐level detection, supporting longitudinal over cross‐sectional interpretation.^28^

Intervening at a molecular subclinical stage raises ethical considerations regarding overtreatment, toxicity and psychological burden. Treating asymptomatic PET‐negative/MRD‐positive patients may medicalize risk, without proven benefit, while withholding treatment may appear to disregard a biological warning. This reflects the MRD's transitional role from research tool to clinical biomarker. A balanced approach requires that MRD positivity identifies vulnerability rather than inevitability and justifies intensified monitoring or trial enrolment rather than routine escalation.

The priority is generating evidence that MRD‐guided strategies improve outcomes.^30^ Early efforts—such as using graft MRD to predict relapse after autologous transplant or ctDNA kinetics after chimeric antigen receptor T‐cell therapy—remain exploratory and unvalidated.^6^

In current practice, MRD is best used to refine prognosis, identify patients for MRD‐guided trials and individualize follow‐up intensity, while avoiding unproven pre‐emptive treatment outside research settings.

## INTEGRATING MRD INTO RESPONSE ASSESSMENT FRAMEWORKS

### Complementarity of metabolic and molecular assessment

Metabolic FDG‐PET primarily assesses local disease control, capturing suppression of glycolytically active tumour aggregates within anatomical compartments, whereas ctDNA‐based MRD reflects systemic clonal burden by detecting persistent malignant lineages regardless of location. Consequently, a patient may achieve PET‐defined control while retaining low‐volume disseminated clones detectable by ctDNA. Conversely, transient metabolic activity may occur despite declining clonal burden, for example due to inflammatory or reparative processes. These biological differences explain the partial concordance between PET and MRD and why their integration improves response classification beyond either modality alone.^7^

Conceptually, integrating PET and MRD shifts response assessment from a unidimensional scale—traditionally framed as complete versus incomplete remission—towards a multidimensional model that incorporates spatial metabolic control with evolutionary clonal extinction. This perspective reflects cancer as a dynamic systemic process rather than a collection of localized lesions and highlights the complementary insights provided by metabolic and molecular data in evaluating treatment response.

### Emerging PET/MRD integrated models

Integrated PET/MRD models define four biological response states: (i) PET‐negative/MRD‐negative, reflecting both anatomical–metabolic control and clonal clearance; (ii) PET‐positive/MRD‐positive, indicating overt treatment failure at both metabolic and molecular levels; (iii) PET‐positive/MRD‐negative, potentially representing inflammatory or treatment‐related metabolic activity despite molecular clearance; and (iv) PET‐negative/MRD‐positive, representing occult residual disease characterized by persistent systemic clonality despite CMR.

**FIGURE 2:**
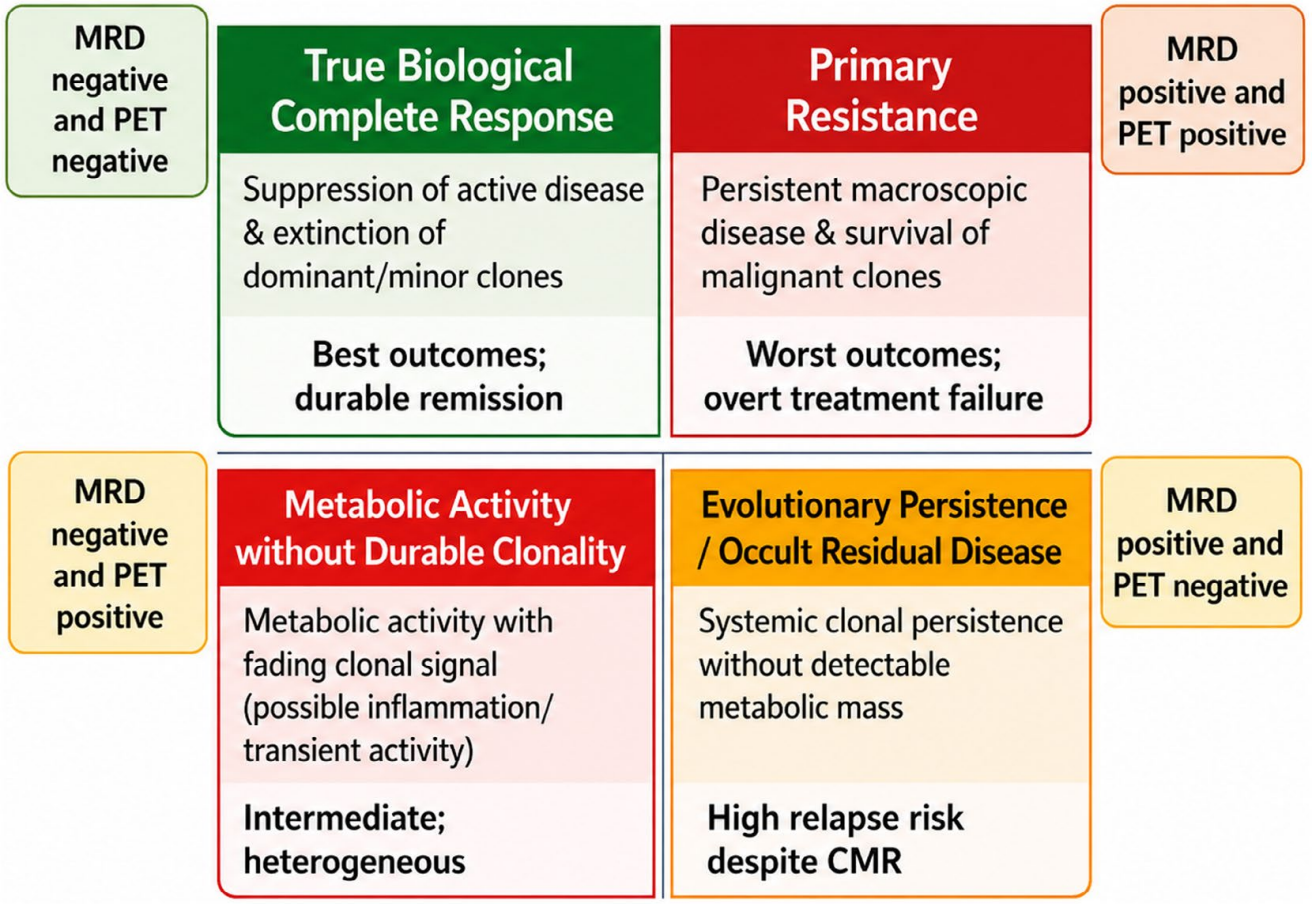
Integrated PET/MRD response categories in DLBCL. DLBCL, diffuse large B‐cell lymphoma; MRD, minimal residual disease; PET, positron emission tomography.

These categories represent distinct biological processes (Figure 2). Concordant categories are relatively straightforward to interpret: PET‐negative/MRD‐negative status implies effective eradication of both dominant and minor clones, whereas PET‐positive/MRD‐positive status indicates resistance at both macroscopic and molecular levels. By contrast, discordant states represent the most clinically challenging scenarios because they reflect dissociation between metabolic activity and systemic clonal persistence. In particular, PET‐negative/MRD‐positive disease reflects evolutionary persistence without tumour reconstruction, while PET‐positive/MRD‐negative patterns may represent transient metabolic activation without sustained clonal maintenance. Combining ctDNA dynamics with PET metrics such as ΔSUVmax improves prognostic discrimination beyond either modality alone. Integrated analyses identify groups with excellent outcomes, intermediate risk and extremely poor PFS despite similar metabolic responses.^6^, ^15^

The main application of PET/MRD integration is within adaptive clinical trial design. Rather than static end‐points, metabolic and molecular responses are increasingly used as dynamic stratifiers capable of modulating trial pathways. Early molecular non‐response in patients who are otherwise PET‐responsive has been proposed to enrich treatment intensification arms, whereas sustained molecular clearance despite equivocal PET findings may justify de‐escalation within experimental protocols.

Both discordant scenarios, therefore, demand careful clinical interpretation and cannot be directly accommodated by conventional response frameworks. Residual FDG uptake after R‐CHOP does not necessarily indicate viable lymphoma, as it may reflect post‐treatment fibrosis, necrosis with reactive inflammation or therapy‐induced immune activation within the tumour microenvironment. These processes can produce persistent or transient PET signals despite undetectable circulating tumour DNA. Thus, PET‐positive/MRD‐negative findings may represent false‐positive metabolic activity rather than true residual disease. Recognizing this limitation is clinically important, as misinterpretation may lead to overtreatment or unnecessary investigations.

The PET‐negative/MRD‐positive scenario represents a clinically relevant discordance that is increasingly recognized as a high‐risk state. Although these patients achieve CMR, persistent ctDNA reflects ongoing systemic clonal survival and is consistently associated with a high likelihood of early relapse. This pattern should therefore not be regarded as a variant of complete response, but rather as evidence of incomplete biological remission. In current practice, it supports closer monitoring and consideration of enrolment in clinical trials, rather than immediate therapeutic intervention.

A limitation of MRD assessment is the biological heterogeneity of residual clones, particularly the distinction between indolent and aggressive populations. In some patients with apparently de novo DLBCL, the aggressive component may arise from transformation of an unrecognized indolent lymphoma. In this setting, MRD positivity at the end of therapy may not indicate persistence of the aggressive disease, but rather survival of an indolent precursor clone following eradication of the aggressive component by immunochemotherapy. As current MRD assays detect tumour‐derived genetic material without distinguishing biological behaviour, interpretation can be challenging. This underscores a key concept: molecular persistence does not always equate to clinically aggressive disease, highlighting the need for studies integrating MRD with clonal architecture and evolutionary dynamics in aggressive lymphomas.

Crucially, these strategies remain hypothesis‐generating rather than practice‐defining. Adaptive designs treat PET/MRD status as a biological signal to test predictive value, not as a directive for routine care. Risk‐adapted strategies aim to determine whether therapy modification based on integrated response improves outcomes. Examples include studies exploring ctDNA‐guided or PET/MRD‐stratified pathways in newly diagnosed DLBCL (NCT03258567, NCT04387239, NCT04863651), and PET‐adapted platforms incorporating molecular sub‐studies exploring discordant responses (NCT03248492). In these settings, PET/MRD integration serves as an experimental variable to enrich biologically defined subgroups and explore therapeutic vulnerability, rather than as a prescriptive algorithm.

Conceptually, these models reflect a shift from retrospective response classification towards response assessment as a prospective experimental variable. They also underscore that the clinical value of MRD lies not in replacing PET but in redefining the biological meaning of ‘response’ by integrating local tumour control with systemic clonal dynamics.

## CONCLUSIONS

Metabolic remission after first‐line therapy in DLBCL does not necessarily correspond to a molecular eradication of the malignant clone. MRD assessment through ctDNA reveals a dimension of residual disease that is invisible to imaging and reflects survival of therapy‐resistant lineages rather than residual tumour bulk. Patients with PET‐negative/MRD‐positive status therefore constitute a biologically distinct subgroup characterized by occult residual disease and a high propensity for relapse.^14^

The complementary nature of PET and MRD reframes response assessment as a multidimensional construct integrating local tumour control with systemic clonal dynamics. While MRD provides independent prognostic information and refines risk stratification among patients achieving complete metabolic responders, its current role remains primarily biological and investigational. At present, the principal arena for PET/MRD integration is adaptive clinical trial design, where combined molecular and metabolic readouts are explored as stratification variables to interrogate response heterogeneity and therapeutic vulnerability.^18^

Accordingly, MRD should not yet be interpreted as a directive for routine therapeutic intervention but rather as a marker of incomplete biological response whose clinical implications require prospective validation. Consistent with recent MRD‐focused reviews, ctDNA‐based MRD in DLBCL can therefore be regarded as a mature prognostic and biological tool but an immature therapeutic biomarker, whose integration into routine clinical decision‐making must await prospective, response‐adapted trials that demonstrate improved patient outcomes.^30^ Ultimately, the value of MRD lies not in replacing PET, but in redefining what ‘response’ means in biological terms. Future frameworks incorporating both metabolic and molecular end‐points may enable a more precise understanding of treatment failure and remission in front‐line DLBCL, provided that such models remain evidence‐driven and are implemented within carefully designed prospective studies.^20^, ^31^

## AUTHOR CONTRIBUTIONS

**Enrica Antonia Martino:** Conceptualization; writing – original draft. **Caterina Labanca:** Conceptualization; writing – original draft. **Santino Caserta:** Conceptualization; writing – original draft. **Ernesto Vigna:** Conceptualization; writing – original draft. **Massimo Gentile:** Conceptualization; writing – original draft. **Antonella Bruzzese:** Conceptualization; writing – original draft. **Eugenio Lucia:** Conceptualization; writing – original draft. **Francesco Mendicino:** Conceptualization; writing – original draft. **Virginia Olivito:** Conceptualization; writing – original draft. **Nicola Amodio:** Conceptualization; writing – original draft. **Fortunato Morabito:** Conceptualization; writing – original draft. **Mamdouh Skafi:** Conceptualization; writing – original draft. **Maria Eugenia Alvaro:** Conceptualization; writing – original draft.

## FUNDING INFORMATION

The authors have nothing to report.

## CONFLICT OF INTEREST STATEMENT

The authors declare that the research was conducted without any commercial or financial relationships that could be construed as a potential conflict of interest.

## Data Availability

Data sharing does not apply to this article, as no new data were generated or analysed in this study.
